# Inbreeding depression is high in a self‐incompatible perennial herb population but absent in a self‐compatible population showing mixed mating

**DOI:** 10.1002/ece3.3354

**Published:** 2017-09-12

**Authors:** Marie Voillemot, John R. Pannell

**Affiliations:** ^1^ Department of Ecology and Evolution University of Lausanne Lausanne Switzerland

**Keywords:** genetic load, heterosis, mating system, outcrossing, purging, selfing

## Abstract

High inbreeding depression is thought to be one of the major factors preventing evolutionary transitions in hermaphroditic plants from self‐incompatibility (SI) and outcrossing toward self‐compatibility (SC) and selfing. However, when selfing does evolve, inbreeding depression can be quickly purged, allowing the evolution of complete self‐fertilization. In contrast, populations that show intermediate selfing rates (a mixed‐mating system) typically show levels of inbreeding depression similar to those in outcrossing species, suggesting that selection against inbreeding might be responsible for preventing the transition toward complete self‐fertilization. By implication, crosses among populations should reveal patterns of heterosis for mixed‐mating populations that are similar to those expected for outcrossing populations. Using hand‐pollination crosses, we compared levels of inbreeding depression and heterosis between populations of *Linaria cavanillesii* (Plantaginaceae), a perennial herb showing contrasting mating systems. The SI population showed high inbreeding depression, whereas the SC population displaying mixed mating showed no inbreeding depression. In contrast, we found that heterosis based on between‐population crosses was similar for SI and SC populations. Our results are consistent with the rapid purging of inbreeding depression in the derived SC population, despite the persistence of mixed mating. However, the maintenance of outcrossing after a transition to SC is inconsistent with the prediction that populations that have purged their inbreeding depression should evolve toward complete selfing, suggesting that the transition to SC in *L. cavanillesii* has been recent. SC in *L. cavanillesii* thus exemplifies a situation in which the mating system is likely not at an equilibrium with inbreeding depression.

## 1 INTRODUCTION

Hermaphrodites can potentially reproduce via a mix of self‐fertilization and outcrossing. Although self‐fertilization allows plants to transmit two copies of their genome to their seed progeny rather than only one, with a substantial potential fitness advantage (Fisher, 1941), the expression of inbreeding depression by selfed progeny (reduced fitness of selfed compared to outcrossed individuals) is thought to constrain the evolution of selfing in hermaphrodite populations (Charlesworth & Charlesworth, 1987; Porcher & Lande, 2005a, 2013; Winn et al., 2011). Selfing is expected to decrease heterozygosity, resulting in the expression of deleterious recessive alleles and an increase in inbreeding depression (Hamrick & Godt, 1996; Wright, Tregenza, & Hosken, 2007). Although selection against inbreeding and the effects of inbreeding depression have likely contributed to the maintenance of various mechanisms to ensure outcrossing, not least self‐incompatibility (Porcher & Lande, 2005b; Sletvold, Mousset, Hagenblad, Hansson, & Ågren, 2013), numerous formally self‐incompatible (SI) species have undergone evolutionary transitions to self‐compatibility (SC) and decreased outcrossing rates (Goodwillie, Kalisz, & Eckert, 2005). These transitions may have been driven by the selective advantage gained by selfing through the transmission of more genes to seed progeny through both ovules and the self‐pollen grains that sire them (automatic transmission advantage; Fisher, 1941) as well as through an ability to reproduce in the absence of mates or pollinators (reproductive assurance advantage; Jain, 1976).

Whatever its cause, a transition from SI to SC and increased self‐fertilization should increase homozygosity in the population and thus the expression of recessive deleterious alleles that would otherwise be protected from purifying selection in heterozygotes, so that mutations that cause inbreeding depression may be purged from the population (Barrett & Charlesworth, 1991; Crnokrak & Barrett, 2002; Dart & Eckert, 2013; Noël et al., 2016). With inbreeding depression purged, selection should more strongly favor the maintenance of self‐fertilization (Lande & Schemske, 1985) and traits that increase its rate and efficiency (Goodwillie et al., 2010). Accordingly, not only do populations that have undergone an evolutionary transition to selfing tend to show substantially reduced inbreeding depression (Husband & Schemske, 1996), but they typically also have smaller flowers than their outcrossing progenitors, with reduced pollen/ovule ratios and reduced nectar production, that is they often display a “selfing syndrome” (Goodwillie et al., 2010; Sicard & Lenhard, 2011).

Intriguingly, not all species that lose an SI system and acquire a capacity to self‐fertilize undergo a transition to full (or nearly full) self‐fertilization and a selfing syndrome. Indeed, many SC species that are derived from SI ancestors continue to outcross to a substantial extent (i.e., they display “mixed mating”): they continue to maintain floral traits in common with outcrossers, with large flowers, copious nectar production, and high pollen/ovule ratios (Dart, Samis, Austen, & Eckert, 2012; Fenster & Martén Rodríguez, 2007). Indeed, of 345 flowering plants species surveyed by Goodwillie et al. (2005), about 40% were estimated to have intermediate selfing rates between 0.2 and 0.8. Although there are numerous models that can explain the maintenance of mixed mating via a number of different mechanisms (reviewed in Goodwillie et al., 2005), mixed‐mating systems continue to be an enigma.

The enigma of mixed mating pertains particularly to patterns of inbreeding depression. In a recent survey of the literature, Winn et al. (2011) found lower values of inbreeding depression for selfing compared to outcrossing taxa, consistent with the theoretical expectation of purging and previous surveys (e.g., Husband & Schemske, 1996), but they also found that inbreeding depression in mixed‐mating populations was similar to that found in fully outcrossing ones. In these species with a high load of deleterious recessive mutations, mixed mating might be interpretable as the result of selection to maintain outcrossing, with selfing as an unavoidable consequence of the pollination mode (e.g., Dart & Eckert, 2013; Kalisz et al., 2012). Such an explanation is, however, inadequate to explain mixed mating in species that have purged their inbreeding depression (e.g., Dart et al., 2012; Kalisz & Vogler, 2003).

In addition to its effects on inbreeding depression, a transition to increased selfing can also affect patterns of heterosis (the increased fitness of offspring resulting from between‐population crosses compared to crosses within populations). Indeed, populations that have purged their inbreeding depression might still maintain substantial genetic load as a result of the fixation of mildly deleterious recessive mutations during a population bottleneck associated with the mating‐system transition (Kirkpatrick & Jarne, 2000), or simply because the effective population size is diminished by increased inbreeding (Roze & Rousset, 2004; Spigler, Theodorou, & Chang, 2016). Moreover, populations that have recently shifted to SC are usually associated with isolation, reduced size and/or increased population differentiation (Duminil, Hardy, & Petit, 2009; Hamrick & Godt, 1996), which can all bring about increased heterosis in between‐population crosses. Such patterns have been found in a number of studies. For instance, Busch (2006) did not find heterosis among five large SI populations and two small SC population of *Leavenworthia alabamica*, but found high levels of heterosis expressed in crosses involving the most isolated self‐fertilizing population. In another recent study, Oakley and Winn (2012) found greater heterosis for small compared to large populations (see also Escobar, Nicot, & David, 2008).

Here, we describe patterns of inbreeding depression and between‐population heterosis for a number of key traits in the long‐lived perennial plant *Linaria cavanillesii*, which shows variation in SI and its mating system, with populations either fully SI, or partially or fully SC (Voillemot & Pannell, 2016). Despite its capacity for autonomous self‐fertilization, the one known SC population maintains a high rate of outcrossing (selfing rate = 0.59) and displays floral traits comparable to that in the SI populations (large floral displays, high nectar production, and high pollen/ovule ratios), suggesting a possible recent loss of SI (Voillemot & Pannell, 2016). We expected to find high inbreeding depression maintained under SI and obligate outcrossing. Moreover, because the SC population does not appear to have undergone a transition toward a selfing syndrome and maintains intermediate to high outcrossing rates, we expected to find high inbreeding depression in this population, too, in line with patterns commonly observed for mixed‐mating species (Winn et al., 2011). Finally, we predicted higher heterosis for crosses involving the SC population compared to SI ones, for example due to the possible fixation of mildly deleterious alleles that may have occurred following a population bottleneck associated with the breakdown of SI and the reduced effective population size under partial inbreeding.

Our study focuses on the single fully SC population of *L. cavanillesii* that we have found in the species’ range. Although this constitutes a narrow base for inference, the evolution of selfing presumably almost always starts in a single population, and inclusion of further populations after its spread from a single point of origin would not broaden the inference base statistically. Our observations contribute to our understanding of the transitions between mating systems in plants more generally by illustrating what is likely a very early stage in the transition from outcrossing to selfing. *L. cavanillesii* is also an outlier in the relation between inbreeding and population isolation and thus serves as a valuable extreme case for studies of mating‐system evolution in plants (Voillemot & Pannell, 2016; and see Discussion). In contrast to the majority of studies reviewed by Winn et al. (2011), in which mixed mating was associated with high inbreeding depression, we found little evidence for any inbreeding depression in the SC population of *L. cavanillesii*, as well as patterns of heterosis that are largely consistent with expectations for populations with a history of high rates of selfing.

## 2 MATERIAL AND METHODS

### 2.1 Site and study species

**Figure 1:**
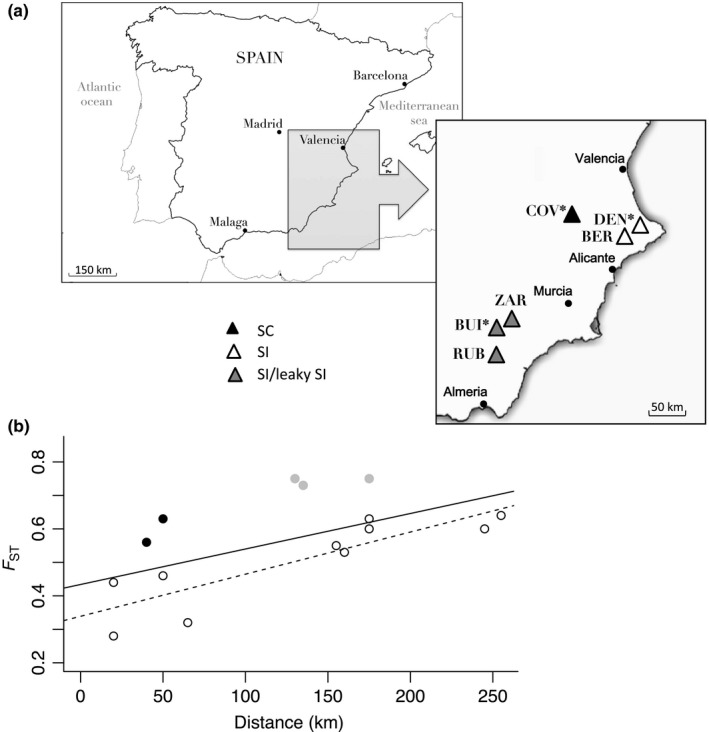
(a) Names and localities of the *Linaria cavanillesii* populations sampled in Spain (SC: self‐compatible population, SI: self‐incompatible population, SI/leaky SI: leaky self‐incompatible population). * indicates populations used for crosses to measure inbreeding depression and heterosis. (b) Pattern of isolation by distance revealed by analysis of microsatellite variation for populations of *L. cavanillesii* across its range, measured in terms of pairwise *F*_ST_. Each dot represents a pair of populations, with gray dots representing distances between SC population and distant populations, and black dots representing distances between SC population and nearby populations. Regression lines are shown for all pairs of populations (full line; Mantel test: *p* = .06) or pairs of SI populations only (dashed line; Mantel test: *p* = .002). Data plotted from Voillemot and Pannell (2016)

*Linaria cavanillesii* is a perennial herb, endemic to southeastern Spain (Laguna et al., 1998), that occurs along north‐northwest‐oriented cliffs at elevations of between 300 m and 1400 m. Flowering occurs between May and June, during which yellow nectar‐spurred flowers are held in large inflorescences that are attractive to pollinators, mainly bees and bumblebees. Around 30 days after fertilization, seeds are dispersed passively from capsules to the wind. See Voillemot and Pannell (2016) for further details. This study was carried out using seeds from three populations in the Alicante‐Murcia region: one fully SC and one leaky SI population as maternal plants, and one additional fully SI population for among population crosses (Figure 1a). Our sampling thus represents the single transition to SC that has been found for *L. cavanillesii* (Voillemot & Pannell, 2016).

### 2.2 Hand‐pollination crosses

To assess and compare inbreeding depression as well as levels of heterosis between the SC and an SI population, we performed controlled crosses in the greenhouses of the University of Lausanne, Switzerland. We used two populations of *L. cavanillesii* as maternal plants: the fully SC population with mixed mating (COV_(SC)_); and a leaky SI population (BUI_(SI)_), which comprises mostly SI individuals as well as some individuals with leaky SI (low production of selfed seeds). One difficulty faced when assessing inbreeding depression for many outcrossing taxa is the difficulty of procuring selfed seed from SI individuals. However, in *L. cavanillesii,* as in other SI species (e.g., Crawford, Archibald, Kelly, Mort, & Santos‐Guerra, 2010; Dart et al., 2012; Zhang, Xiong, & Huang, 2014), the occasional production of selfed seeds is common, a phenomenon known as pseudoself‐compatibility or leaky SI (Levin, 1996). In this study, we obtained selfed progeny from seeds produced via pseudoself‐compatibility for the SI population. A second SI population (DEN_(SI)_) was used as donor plants to compare levels of heterosis (see Figure 1a).

We applied four treatments: “self‐pollination” (inflorescence bagged at the bud stage, anthers removed prior to anthesis, and pollination with self‐pollen); “outcrossing within population” (inflorescence bagged at the bud stage, anthers removed prior to anthesis, and pollination with outcross pollen from a different plant of the same population); two “outcrossing between populations” (inflorescence bagged at bud stage, anthers removed prior to anthesis, and fertilization with outcross pollen from a plant coming from a different population, either DEN_(SI)_, BUI_(SI)_ or COV_(SC)_, depending on the treatment). When describing crosses, the first population name represents the maternal plant, and the second one the pollen donor. Crosses within and between populations were used to estimate heterosis. Heterosis was compared between (i) SC vs. SI populations from crosses with geographically distant SI populations (crosses COV_(SC)_—BUI_(SI)_ and BUI_(SI)_—DEN_(SI)_, respectively); (ii) geographically distant vs. nearby SI populations from crosses with the SC population (crosses COV_(SC)_—BUI_(SI)_ and COV_(SC)_—DEN_(SI)_, respectively).

The data we present are derived from a total of 82 crosses using nine and thirteen mother plants from the BUI_(SI)_ (40 crosses) and COV_(SC)_ populations (42 crosses), respectively, and our results are based on several traits measured on a total of 208 progeny plants raised to maturity (98 for COV_(SC)_ and 110 for BUI_(SI)_; see Table S1 for details). Our sampling was limited by difficulties we faced in raising plants from seeds (see Discussion), but our analysis nevertheless allows us to draw several clear conclusions.

To exclude potential pollinators, we bagged inflorescences with small fine‐meshed nylon bags a few days before flowers opened, and we marked individual flowers with colored string corresponding to each treatment. We removed anthers carefully with fine forceps at the same time. After 3 days (when stigmas were receptive), we applied appropriate pollen, depending on treatment. Self‐pollen was taken from intact flowers on the same plant, whereas outcross pollen came from another plant from the same or from a different population. We applied pollen by gently brushing an anther against a target stigma until it was fully covered with pollen. After 14 days, we investigated any successful fertilization by observing fruit formation. We applied rubber glue to the apex of developing fruit capsules after 18 days, when fruits were fully formed, to prevent seed loss before collection. We collected fruits approximately 10 days after they had been glued to ensure that seeds were fully mature.

### 2.3 Progeny phenotype and fitness measurements

To estimate different phenotypic and fitness‐related traits, plants were grown in a greenhouse of the University of Lausanne. We counted and weighed all seeds for each cross, then sowed ten seeds from each fruit in Petri dishes placed in a phytotron (conditions: 13 hr days, 20°C:15°C day:night, 80% humidity) and assessed germination rates, accordingly. We then transplanted four randomly chosen seedlings into larger pots, noting seedling size at the time of transplantation. After transplantation, all plants were placed on glasshouse benches in a random block design. Additionally, we moved each table and randomized plants within the table once a week. We measured growth 9 weeks after transplantation (difference in size compared with the initial transplantation size), and recorded the number of days from transplantation to flowering, as well as flower production, over a period of 3 months. We also measured phenotypic traits, including flower longevity, flower size, pollen production, ovule number, pollen/ovule ratio, and nectar quantity and quality (measured with two refractometers to cover full range of nectar concentration: 0–50 Brix; and 45–80 Brix; Bellingham & Stanley Ltd, Tunbridge Wells, UK). To account for flower variability within plants, we took and averaged every measure of flowers using at least three flowers per plant.

We used a particle counter (Elzone II 5390 Micromeritics^®^) to estimate the number of pollen grains. For each sample, one upper and one lower anther of a nonopened flower were fixed in formaldehyde‐acetic acid alcohol solution (FAA; 5 parts glacial acetic acid: 5 parts 38% formaldehyde: 90 parts 70% ethanol). Before analysis, samples were sonicated (Branson 52, Emerson industrial automation) for two minutes to release pollen from anthers, and then transferred into the analysis beaker of the particle counter, which contained 100 ml of ddH_2_O with 2% NaCl. Each sample was analyzed four times for 30 s, and the average of the four replicates was used to estimate the number of pollen grains per anther. We estimated pollen production for each plant as the average over measures taken for three flowers per plant. We estimated the number of ovules for the same flowers used for pollen counting, based on floral dissections using a razor blade, and counted under a binocular microscope (Leica MZ 125, Leica Microsystems^®^).

### 2.4 Data analysis

All analysis was conducted in R (version 3.1.2 or higher, R Core Team 2015). We analyzed seed production and seed mass resulting from initial crosses by analysis of variance of per‐family averages, using Tukey post hoc tests for multiple comparisons of means. For most other traits, we analyzed the effect of crosses for each population with linear mixed models, using the lme4 package in R (Bates, Mächler, Bolker, & Walker, 2014), and setting pollination treatment as a fixed factor and block and mother as nested random factors. For the assessment of phenotypic differences, we included the timing of measurement as an additional random factor. For nectar measurements, we included temperature and humidity as random factors. We analyzed significance of variables through a stepwise deletion procedure; in the final model, multiple comparisons of means were performed using the lmerTest package in R (Kuznetsova, Brockhoff, & Christensen, 2014). For binomial data such as the proportion of seed germination, we used a generalized mixed model (glmer), with maternal plant identity treated as a random factor and data modeled as binomial. If significant, differences within treatments were then tested by means of post hoc tests, with the glht function of the multcomp package in R. All statistical results are summarized in Table 1.

**Table 1:** Summary of statistical results for all the traits measured for 208 plants (nested within 52 maternal families), for one self‐compatible (SC) and one self‐incompatible (SI) population and resulting from hand self‐, and cross‐fertilization within and among populations

Traits	COV (SC)			BUI (SI)		
	*df*	*F*	*p*‐Value	*df*	*F*	*p*‐Value
Seed number*	3, 24.0	2.59	.08	3, 18.0	4.41	.02
Seed weight*	3, 23.0	1.57	.22	3, 18.0	1.08	.38
Proportion germination	3	39.42#	<.001	3	15.59#	.001
Days to flowering	3, 77.0	0.30	.83	3, 56.3	23.59	<.001
Flower production	3, 80.2	1.14	.34	3, 39.2	1.36	.27
Growth after nine weeks	3, 81.5	6.43	<.001	3, 81.2	5.95	.001
Flower size	3, 122.0	4.75	.004	3, 96.0	2.67	.05
Nectar quantity	3, 100.5	0.60	.61	3, 59.1	5.56	.002
Nectar quality	3, 108.5	8.28	<.001	3, 79.8	1.40	.25
Flower longevity	3, 59.0	0.97	.41	3, 45.9	3.17	.03
Pollen production	3, 76.9	7.81	<.001	3, 23.0	1.45	.25
Ovule production	3, 45.1	1.75	.17	3, 42.9	0.45	.72
Pollen/ovule ratio	3, 23.8	5.70	.004	3, 19.3	1.70	.2

Number of degrees of freedom (*df*), *F*‐ratios, and *p*‐values are reported. * indicates traits that have been analyzed using analysis of variances. Proportion of germination was analyzed using a generalized mixed model (# represents *chi‐square* test instead of *F*‐ratios), all other traits were analyzed using random linear models, with block and family treated as random factors. For growth, flower size and nectar measurement, group, temperature, and hygrometry were added as random factors (see text for details). Significant results are highlighted in gray.

### 2.5 Estimates of heterosis and inbreeding depression

Following Ågren and Schemske (1993), we calculated inbreeding depression as 1—(w_s_/w_ow_) when selfed offspring had lower trait values than outcrossed progeny, and as (w_ow_/w_s_)—1 otherwise; where w_s_ and w_ow_ were per‐family mean trait values for selfed and outcrossed within‐population treatments, respectively. Values of inbreeding depression were thus scored on a scale from −1.0 to 1.0, with larger values indicating higher levels of performance, except for time to flowering, which we inversed for interpretation. Heterosis was calculated as 1−(w_ow_/w_ob_), where w_ow_ and w_ob_ are per‐family mean trait values in outcrossed within population and outcrossed between‐population treatments, respectively. Positive heterosis indicates higher levels of performance of progeny resulting from between‐population crosses, whereas negative heterosis indicates outbreeding depression.

Most measured traits are phenotypic traits (e.g., nectar production), but some traits are more directly related to fitness. Thus to estimate a composite measure of early‐acting inbreeding depression based on fitness‐related traits only, we computed an average cumulative index of fitness based on the product of seed production (but see Discussion for an estimation of fitness without seed production), proportion of seed germination, plant growth, number of flowers produced at the time of harvest and an estimate of the number of pollen grains and ovules produced per flower. Because *L. cavanillesii* is a long‐lived perennial herb that probably lives for many years, it was not possible to estimate components of inbreeding depression expressed in older adult plants (see Discussion).

## 3 RESULTS

### 3.1 Measures of inbreeding depression

**Figure 2:**
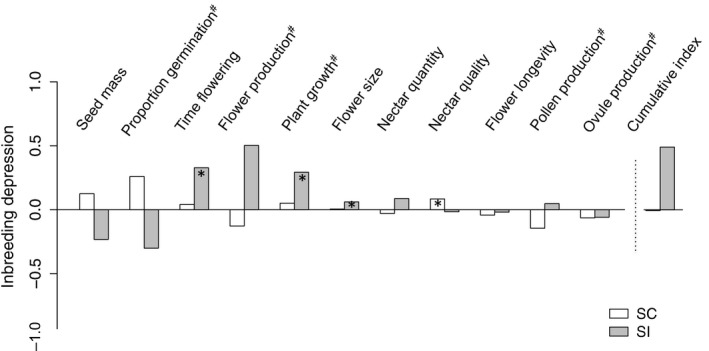
Comparison of inbreeding depression for different phenotypic traits in one self‐compatible (SC) population and one self‐incompatible (SI) population of *Linaria cavanillesii*. Values plotted were calculated as the means of per‐family estimates of inbreeding depression. The cumulative fitness index combines the product of traits indicated with a ^#^: proportion of seed germination, plant growth, number of flowers produced at the time of harvest, and average of pollen and ovule production per flower. * indicates significant results after post hoc tests (*p* < .05)

Considering all the 13 variables investigated, inbreeding depression for the SC population was significant only for nectar quality (Table 1 and Figure 2;Figs. S1–S7). In contrast, the SI population showed significant inbreeding depression for four of the traits measured (seed production, days to flowering, growth after 9 weeks and flower size; Table 1 and Figure 2; Figs. S1–S7). The average cumulative fitness index gave an inbreeding depression value of 0.00 for the SC population and 0.85 (including measures for seed production) or 0.49 (excluding seed production; see Discussion) for the SI population (Figure 2).

### 3.2 Measures of heterosis

**Figure 3:**
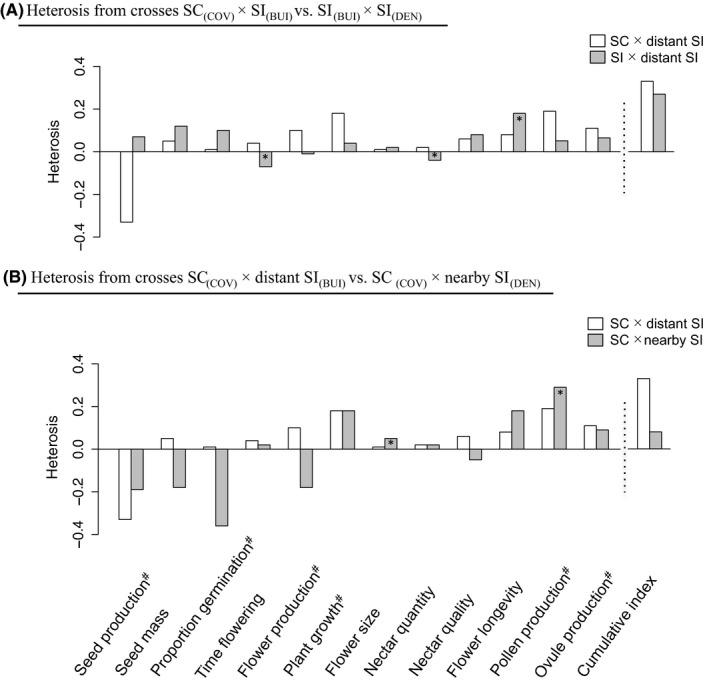
Estimates of heterosis revealed by crosses between different populations of *Linaria cavanillesii*. The first term describing the crosses corresponds to the maternal plant and the second one to the pollen donor. (a) Heterosis revealed for crosses between geographically distant populations, where one population in the cross was SC (white; DEN_(_ _SI__)_—COV_(_ _SC__)_) or both populations were SI (gray; DEN_(_ _SI__)_—BUI_(_ _SI__)_). (b) Heterosis revealed for crosses involving the SC population with a distant SI population (white; COV_(_ _SC__)_—BUI_(_ _SI__)_) or a nearby SI population (gray; COV_(_ _SC__)_—DEN_(_ _SI__)_). Plotted values were calculated as the means of per‐family estimates of heterosis. The cumulative fitness index combines the product of traits indicated with a ^#^: seed production, proportion of seed germination, plant growth, number of flowers produced at the time of harvest, and average of pollen and ovule production per flower. * indicates significant results after post hoc tests (*p* < .05)

Our results revealed heterosis for both the SI and the SC populations (Figure 3; Figs. S1–S7). Except for seed production, every trait measured in the SC population showed a greater value when plants were crossed with plants from another more distant population (Figure 3). For the SI population, only time to flowering, nectar quantity, and flower longevity showed any evidence for significant differences between crosses (Figure 3). The average cumulative fitness index gave a heterosis value of 0.33 for the SC population and 0.27 for the SI population.

For crosses involving the SC population, we found a lower heterosis when crosses were performed with the nearby population (Figure 3). Among the 13 traits measured, only flower size and pollen production showed significant heterosis for crosses with a nearby population (Table 1; Figure 3 and Figs. S4 and S6). The average cumulative fitness index yielded a heterosis value of 0.08 compared to 0.33 for distant population crosses.

## 4 DISCUSSION

Our study revealed high inbreeding depression (ID) for self‐fertilized individuals from a self‐incompatible (SI) population of *Linaria cavanillesii* (for days to flowering, growth, flower production, and flower size), as expected for an outcrossing perennial population (Winn et al., 2011). In contrast, we found little evidence for ID for crosses of a self‐compatible (SC) population that displays mixed mating. In both populations, substantial heterosis was found for crosses between more distant populations, but crosses between nearby populations involving the SC population resulted in lower heterosis.

### 4.1 High inbreeding depression in a self‐incompatible population

Intrapopulation crosses revealed significant ID for the SI population, especially for time to flowering and flower production. We also found that selfing yielded lower numbers of seeds than outcrossing. Because we estimated seed production on the basis of the spontaneous production of seeds by leaky SI individuals, this result is likely to be partly due to SI rather than ID. Nevertheless, the presence of seeds with an abnormal size (likely to be aborted) in selfed fruits suggests that ID may have affected early seed development, too. Calculating a combined index of ID across all stages measured, ignoring seed set, ID for early stages of growth and reproduction of young adults in outcrossing populations of *L. cavanillesii* was δ = 0.49. If the difference in seed set between selfed and outcrossed progeny were fully attributed to ID, this estimate would increase substantially to δ = 0.85. It seems likely that δ for outcrossing populations of *L. cavanillesii* lies between these two values, not least because we measured components of fitness for only young plants in a perennial species in which individuals may live for many years. It is thus plausible that δ > 0.5 for outcrossing populations of *L. cavanillesii*.

An estimate of δ > 0.5 is consistent with a scenario in which outcrossing should be maintained by selection (Lande & Schemske, 1985), and it is in broad agreement with other empirical studies; for example in a review of estimates of ID for 58 outcrossing plant species, Winn et al. (2011) calculated an average lifetime of δ = 0.54. More recently, a study on the perennial *Arabidopsis lyrata* revealed that two of its SI populations showed high ID (Sletvold et al., 2013; but see Willi, 2013). It would thus seem that ID is an important force preventing the selective loss SI or the spread of SC in populations of *L. cavanillesii*. The fact that almost all populations of *L. cavanillesii* surveyed have a low proportion of individuals with leaky SI (partial SC; Voillemot & Pannell, 2016) suggests that SC should be able to spread if it were advantageous. The maintenance of SI across most of the species’ range suggests that SC is probably disadvantageous in these populations, consistent with our inferences for inbreeding depression.

### 4.2 Low inbreeding depression in a mixed‐mating population

We found little evidence for ID in the SC mixed‐mating population, whether measured at early or later stages of early adult growth (average cumulative δ = 0.0). Recall that this mixed‐mating population shows relatively high levels of outcrossing (selfing rate = 0.59; Voillemot & Pannell, 2016), despite the ability of its individuals to self autonomously (because anthers are in contact with stigmas). The absence of ID is thus somewhat surprising, as other mixed‐mating species tend to harbor similar levels of ID as SI species (Winn et al., 2011). Rather, our result is more in line with expectations for highly selfing species, in which ID is quickly purged (Barrett & Charlesworth, 1991; Busch, 2005; Noël et al., 2016). The observed patterns of mating and lack of ID in the SC population of *L. cavanillesii* thus pose the intriguing question as to why outcrossing persists in the face of the expected automatic selection of increased selfing (Fisher, 1941). Although this pattern is unusual, Goodwillie et al. (2005) found in their survey that ID was negative for seven species out of 64 species showing mixed mating (i.e., 0.2 < selfing rate < 0.8), and Winn et al. (2011) identified four mixed‐mating species out of 38 that showed reduced ID (i.e., δ < 0.3), with one showing negative ID (*Collinsia heterophylla*, δ = −0.37). Even if some of these species are in a state of evolutionary transition toward higher selfing rates (e.g., Dart & Eckert, 2013; Goodwillie, 2000; Goodwillie, Partis, & West, 2004), it is still not clear what might stabilize others.

Several hypotheses are suggested to explain how mixed mating could be stable. Holsinger (1991) showed that pollen discounting might maintain mixed mating, predicting that in a highly selfing population, an individual exporting pollen would always have a transmission advantage, therefore allowing mixed mating to become stable. This has been observed in *Ipomoea purpurea* for instance, where frequency‐dependent pollen discounting has indeed been shown to maintain mixed mating (Chang & Rausher, 1998). This result was confirmed by more complicated models including variation in ID and pollen limitation, even if low stable intermediate selfing rates were also shown to result from unavoidable geitonogamy (Porcher & Lande, 2005a). Mixed mating might also be stable if it provides reproductive assurance in cases of spatiotemporal variation in pollinator services. In *Collinsia verna*, for instance, variability in pollinator environment may be sufficient to maintain substantial outcrossing rates despite low ID (Kalisz & Vogler, 2003, 2004). However, in most cases, the maintenance of outcrossing in species showing an absence of ID still remains puzzling and unexplained.

There would seem to be at least three possible explanations for the observed pattern of mixed mating and lack of ID in the SC population of *L. cavanillesii*. First, if the loss of SI were associated with the colonization of a new population involving a strong bottleneck, then the newly founded population may have low genetic diversity from the outset. Although such a population might express high genetic load (Kirkpatrick & Jarne, 2000), the low genetic diversity at viability loci could persist following an increase in population size in the absence of further migration, so that ID would remain low. This sort of explanation has been suggested to explain the loss of SI in the North American populations of *Arabidopsis lyrata* in which ID may have been purged via a population bottleneck during the colonization of North America (Foxe et al., 2010; but see Oakley, Spoelhof, & Schemske, 2015). Similarly, in *Leavenworthia alabamica*, newly selfing populations showed reduced ID, probably as a consequence of purging during establishment after long‐distance colonization through seed dispersal (Busch, 2005), an explanation consistent with the finding of strong heterosis in the most geographically isolated and divergent selfing population (Busch, 2006). As discussed below, however, our own inferences concerning heterosis are not entirely consistent with this idea.

Second, it is possible that we have underestimated ID in the SC population of *L. cavanillesii*. We measured ID in relatively small families, and only for early‐stage components for the life cycle of a relatively long‐lived perennial species, in which ID might occur at later (unmeasured) stages (e.g., Lobo, Jiménez, Solís‐Hernandez, & Fuchs, 2015). Indeed, genetic load expressed in early life‐history stages may be purged more easily than that in later life‐history stages (Husband & Schemske, 1996). We also measured traits on plants growing in the greenhouse, likely a benign environment in which ID may be less strongly expressed (reviewed in Armbruster & Reed, 2005). Under field conditions, inbreeding depression might, for example, be enhanced by predation or parasite pressure sufficiently to disfavor a shift to complete selfing (Agrawal & Lively, 2001; Campbell, 2014; Carr & Eubanks, 2014). Seed predation in natural populations of *L. cavanillesii* is severe, with up to 50% of its seeds lost to seed‐predating weevils (M. Voillemot and J.R. Pannell, personal observations). If weevils preferentially eat the seeds of inbred individuals (e.g., Bello‐Bedoy & Cruz L, 2011), ID in the field might actually be substantially higher than our estimate. Nevertheless, while we cannot directly rule out these possibilities, inbreeding depression at later life stages should restore the population toward Hardy–Weinberg equilibrium (Ritland, 1990), yet adult plants of *L. cavanillesii* in the SC population continue to display high inbreeding coefficients in the field (*F*_*IS*_ = 0.36; Voillemot & Pannell, 2016). It thus seems unlikely that our estimates of low inbreeding depression are qualitatively inaccurate. Recall that ID was measured under the same conditions for the SI and SC population, and that putative effects of a benign environment did not prevent the expression of ID by plants from the SI population.

Third, the transition to SC in *L. cavanillesii* may have been so recent that the SC population has simply not had time to respond to selection for an increased selfing rate. Under this scenario, mixed mating may reflect a transitional state toward increased selfing rates. Some aspects indeed point to a recent divergence between the SI and SC populations of *L. cavanillesii* (Voillemot & Pannell, 2016). In particular, we did not observe any phenotypic changes in flower morphology toward a selfing syndrome, which one might have expected for a population that has lost SI (Voillemot & Pannell, 2016). A similar scenario was suggested for *Leavenworthia alabamica* (Busch, Joly, & Schoen, 2011) and *Arabidopsis lyrata* (Hoebe, 2009), both species in which a recent loss of SI was given as a possible explanation for the absence of a selfing syndrome in SC populations. Similarly, the occurrence of outcrossing despite negligible ID in some populations of *Camissoniopsis cheiranthifolia* might be due to the fact that they are still in the process of evolving higher levels of selfing (Dart & Eckert, 2013).

### 4.3 Variation in heterosis for self‐compatible vs. incompatible populations

Our study has found evidence for heterosis expressed in crosses between geographically relatively distant populations, for crosses involving both the SC and SI populations. Except for seed production, every trait measured in the SC population showed greater values when plants were crossed with those from a distant population. Crosses involving only SI populations also revealed heterosis, but somewhat less so overall (e.g., heterosis was not apparent for time to flowering or nectar quantity). These results are broadly consistent with expectations based on patterns of population differentiation (Figure 1b). In particular, while *L. cavanillesii* shows a clear signal of genetic isolation by distance for both SI and SC populations, population differentiation tends to be higher for pairs of populations involving the SC population (Figure 1b). Interestingly, we found no evidence for significant overall heterosis expressed in crosses between populations involving the SC population and sampled in close proximity (average cumulative index of heterosis = 0.08). Here, only pollen production and plant growth showed evidence for any significant degree of heterosis. This result, too, is consistent with the pattern of isolation by distance found for *L. cavanillesii*. It suggests either that the shift from SI to SC has not erased this pattern or that there has been continued migration among nearby populations that differ in their SI status. Again, these patterns recall those found by Busch (2006) for *Leavenworthia alabamica*, where strong reproductive isolation of one SC population resulted in high heterosis in crosses with other populations, whereas another more connected SC population did not. Taken together, our results suggest that the mating system alone is not sufficient to account for differences in heterosis between populations but that population size or isolation is important, too (Oakley & Winn, 2012; Oakley et al., 2015).

### 4.4 Concluding remarks

Theoretical and empirical work point to a change in ID as one of the main forces influencing the transition to selfing and the apparent stability of mixed mating (Charlesworth & Charlesworth, 1987; Winn et al., 2011). Our observation of high ID for SI individuals of *L. cavanillesii* is consistent with the idea that selection against inbreeding in these populations might indeed prevent the spread of mutations conferring a capacity to self‐fertilize in these populations. In contrast, our observations for the mixed‐mating population run counter to our expectations based on findings in other studies (Winn et al., 2011). It appears that mutations that might cause ID have largely been purged from the SC population, despite the fact that outcrossing rates continue to be high due to high pollinator visitation rates. It remains possible that *L. cavanillesii* continues to be selected for outcrossing and the maintenance of an outcrossing floral syndrome under variable pollinator availability, as has been suggested for other species (e.g., Dart et al., 2012; Kalisz & Vogler, 2003, 2004). Nevertheless, the selection of expensive traits that maintain outcrossing is difficult to reconcile with the absence of ID observed in the SC population of *L. cavanillesii*. Ultimately, mixed mating in *L. cavanillesii* is more likely to be an outcome of a recent loss of SI, and the failure of natural selection, through lack of time and/or relevant genetic variation, to bring about a shift toward complete selfing and a selfing syndrome.

## CONFLICT OF INTEREST

The authors declare no conflict of interest.

## AUTHORS CONTRIBUTION

MV and JRP conceived and designed the experiments. MV performed the experiment, collected, and analyzed the data. MV and JRP contributed to writing, revising, and approving the final draft of the manuscript.

## DATA ACCESSIBILITY

Data analyzed in this study are available on the Dryad Digital Repository: https://doi.org/10.5061/dryad.4gc16.
