# Supplementary material for: Inbreeding depression is high in a self‐incompatible perennial herb population but absent in a self‐compatible population showing mixed mating
Source: Ecol Evol. 2017 Sep 12;7(20):8535–44. doi: 10.1002/ece3.3354 (PMC5648656; doi:10.1002/ece3.3354)
Supplement: Supplementary file 1 [file ECE3-7-8535-s001.doc]

Supplementary information

**Table S1.** Summary of sampling design to estimate inbreeding depression in *Linaria cavanillesii*. Number of families and number of plants assayed per family are shown for each treatment. Treatments were: self (self pollen), outcross-within (outcross pollen from a plant within population), outcross-between (pollen from another population, either DEN_(SI)_, COV_(SC)_, or BUI_(SI)_).

**Figure S1**. **Comparison of (a) seed production and (b) seed mass, between two populations of *Linaria cavanillesii* following different crossing treatments** (SC: self-compatible; SI: self-incompatible; out_b: outcross between populations; out_w: outcross within population). Significant differences are indicated with no letters in common; bars indicate mean ± SEM. Number of replicates is indicated beneath each data point.

**Figure S2.** **Comparison of (a) number of days from germination to flowering and (b) total flower production overs three months, between two populations of *Linaria cavanillesii* following different crossing treatments** (SC: self-compatible; SI: self-incompatible; out_b: outcross between populations; out_w: outcross within population). Significant differences are indicated with no letters in common; bars indicate mean ± SEM. Number of replicates is indicated beneath each data point.

**Figure S3.** **Comparison of growth (difference with initial transplantation size) nine weeks after transplanting between two populations of *Linaria cavanillesii* following different crossing treatments** (SC: self-compatible; SI: self-incompatible; out_b: outcross between populations; out_w: outcross within population). Significant differences are indicated with no letters in common; bars indicate mean ± SEM. Number of replicates is indicated beneath each data point.

**Figure S4.** **Comparison of (a) flower size and (b) flower longevity, between two populations of *Linaria cavanillesii* following different crossing treatments** (SC: self-compatible; SI: self-incompatible; out_b: outcross between populations; out_w: outcross within population). Significant differences are indicated with no letters in common; bars indicate mean ± SEM. Number of replicates is indicated beneath each data point.

**Figure S5**. **Comparison of (a) nectar quantity and (b) nectar quality, between two populations of *Linaria cavanillesii* following different crossing treatments** (SC: self-compatible; SI: self-incompatible; out_b: outcross between populations; out_w: outcross within population). Nectar quantity was measured as the length of spur showing nectar. Significant differences are indicated with no letters in common; bars indicate mean ± SEM. Number of replicates is indicated beneath each data point.

**Figure S6. Comparison of (a) ovule number and (b) pollen grains production, between two populations of *Linaria cavanillesii* following different crossing treatments** (SC: self-compatible; SI: self-incompatible; out_b: outcross between populations; out_w: outcross within population). Significant differences are indicated with no letters in common; bars indicate mean ± SEM. Number of replicates is indicated beneath each data point.

**Figure S7. Comparison of pollen/ovule ratio between two populations of *Linaria cavanillesii* following different crossing treatments** (SC: self-compatible; SI: self-incompatible; out_b: outcross between populations; out_w: outcross within population). Significant differences are indicated with no letters in common; bars indicate mean ± SEM. Number of replicates is indicated beneath each data point.

Table S1.

|  | **Treatment** | **Number of families** | **Number of plants assayed** | |  |  | **Treatment** | **Number of families** | **Number of plants assayed** | |
| --- | --- | --- | --- | --- | --- | --- | --- | --- | --- | --- |
|  |  |  |  |  | |  |  |  | |  |
| **BUI (SI)** | Self | 3 | 11 |  |  | **COV (SC)** | Self | 10 | | 42 |
|  | Outcross between (COV_(SC)_) | 7 | 27 |  | |  | Outcross between (BUI_(SI)_) | 8 | | 35 |
|  | Outcross between (DEN_(SI)_) | 6 | 30 |  | |  | Outcross between (DEN_(SI)_) | 6 | | 18 |
|  | Outcross within | 6 | 30 |  | |  | Outcross within | 4 | | 15 |
|  |  |  |  |  |  |  |  |  |  |  |
| Total |  |  | 98 |  | |  |  |  | | 110 |

**Figure S1**

**Figure S2**

**Figure S3**

**Figure S4**

**Figure S5**

**Figure S6**

**Figure S7**
